# Stat3 Downstream Gene Product Chitinase 3-Like 1 Is a Potential Biomarker of Inflammation-induced Lung Cancer in Multiple Mouse Lung Tumor Models and Humans

**DOI:** 10.1371/journal.pone.0061984

**Published:** 2013-04-22

**Authors:** Cong Yan, Xinchun Ding, Lingyan Wu, Menggang Yu, Peng Qu, Hong Du

**Affiliations:** 1 Department of Pathology and Laboratory Medicine, Indiana University School of Medicine, Indianapolis, Indiana, United States of America; 2 The Center for Immunobiology, Indiana University School of Medicine, Indianapolis, Indiana, United States of America; 3 IU Simon Cancer Center, Indiana University School of Medicine, Indianapolis, Indiana, United States of America; 4 Department of Biostatistics & Medical Informatics, University of Wisconsin-Madison, Madison, Wisconsin, United States of America; Shanghai Jiao Tong University School of Medicine, China

## Abstract

Over-activation of the signal transducers and activators of the transcription 3 (Stat3) pathway in lung alveolar type II (AT II) epithelial cells induces chronic inflammation and adenocarcinoma in the lung of CCSP-rtTA/(tetO)_7_-CMV-Stat3C bitransgenic mice. One of Stat3 downstream genes products, chitinase 3-like 1 (CHI3L1) protein, showed increased concentration in both bronchioalveolar lavage fluid (BALF) and blood of doxycycline-treated CCSP-rtTA/(tetO)_7_-CMV-Stat3C bitransgenic mice. When tested in other inflammation-induced lung cancer mouse models, the CHI3L1 protein concentration was also highly increased in BALF and blood of these models with tumors. Immunohistochemical staining showed strong staining of CHI3L1 protein around tumor areas in these mouse models. Analysis of normal objects and lung cancer patients revealed a significant elevation of CHI3L1 protein concentration in human serum samples from all categories of lung cancers. Furthermore, recombinant CHI3L protein stimulated proliferation and growth of Lewis lung cancer cells. Therefore, secretory CHI3L1 plays an important role in inflammation-induced lung cancer formation and potentially serve as a biomarker for lung cancer prediction. Based on our previous publication and this work, this is the first animal study linking overexpression of CHI3L1 to various lung tumor mouse models. These models will facilitate identification of additional biomarkers to predict and verify lung cancer under various pathogenic conditions, which normally cannot be done in humans.

## Introduction

Lung cancer is the most deadly cause in human cancer population. The five year survival rate is only about 15%. There is an urgent need for identifying biomarkers that can be used to predict lung cancer occurrence. Recently, we demonstrated that both lung epithelial cell-initiated regional inflammation and myeloid cell-initiated systemic inflammation can induce spontaneous lung tumorigenesis in multiple lung tumor animal models [1], [2], [3], [4], [5], indicating that inflammatory molecules are potentially useful for lung cancer prediction. These inflammation-induced lung tumor animal models are ideal and valuable systems for identification and verification of lung cancer biomarkers. During the pathogenic process of chronic inflammation and lung tumorigenesis in these animal models, a common feature is Stat3 over-activation in inflammatory myeloid derived suppressive cells (MDSCs) and lung epithelial cells, suggesting that Stat3 plays a critical role in inflammation-induced lung tumorigenesis. Indeed, when a constitutive active form of Stat3C is over-expressed in alveolar type II epithelial cells, it induces downstream inflammatory genes. Subsequently, up-regulation of these inflammatory molecules stimulates and recruits inflammatory cells (such MDSCs) into the lung to form an inflammatory environment. Persistent presence of these inflammatory cells facilitates spontaneous adenocarcinoma in the lung of CCSP-rtTA/(tetO)_7_Stat3C bitransgenic mice [1], [6].

Based on these observations, we hypothesize that Stat3 downstream genes serve as potential biomarkers for inflammation-induced lung cancer prediction. Affymetrix GeneChip microarray analysis reveals around 800 Stat3 downstream genes in the lung of CCSP-rtTA/(tetO)_7_Stat3C bitransgenic mice as we reported previously [1]. When tested in animals and humans, some of these genes can be used as biomarkers for lung cancer [1], [7]. Since most of these genes are intracellular proteins, it is difficult to use them for the purpose of clinical diagnosis and prognosis without going through biopsy. Therefore, there is a need to identify soluble and secretory proteins as biomarkers in serum or bronchioalveolar lavage fluid (BALF) samples for lung cancer prediction and verification. Here, we report that secretory protein Chitinase 3-Like 1 (CHI3L1), a Stat3 downstream gene product, is a potential biomarker for lung cancer prediction in various animal tumor models and humans.

## Methods

### Animal Care

All scientific protocols involving the use of animals have been approved by the Institutional Animal Care and Use Committee (IACUC) of Indiana University School of Medicine and followed guidelines established by the Panel on Euthanasia of the American Veterinary Medical Association. Protocols involving the use of recombinant DNA or biohazardous materials have been approved by the Biosafety Committee of Indiana University School of Medicine and followed guidelines established by the National Institutes of Health. Animals were housed under (IACUC)–approved conditions in a secure animal facility at Indiana University School of Medicine. All the cell specific transgenic mice have been previously described [1], [2], [3], [4], [5].

### ELISA

For mouse serum collection, the abdominal cavities of doxycycline-treated or untreated CCSP-rtTA/(TetO)_7_-CMV-Stat3, CCSP-rtTA/(TetO)_7_-CMV-MMP12, CCSP-rtTA/(TetO)_7_-CMV-Api6, c-fms-rtTA/(TetO)_7_-CMV-Api6 and c-fms-rtTA/(TetO)_7_-CMV-MMP12 bitransgenic mice were opened after anesthetizing with triple sedative by intraperitoneal (IP) injection. The mouse blood samples were collected from interior vena cava (IVC). Sera were separated by centrifugation at 1,500 rpm for 10 minutes at 4°C. For bronchioalveolar lavage fluid (BALF) collection, the trachea was isolated and cannulated with a 20 gauge luer stub adapter**.** Using a 1 cc syringe, bronchioalveolar lavage fluid (BALF) was collected by perfusing the lung with 1 ml aliquot of 0.9% sodium chloride and withdrawing back fluids. BAL fluids were centrifuged for 5 minutes at 1,000 rpm and 4°C to remove cell pellets. CHI3L1 concentrations in mouse and human serum (50–100 µl) were determined by Mouse Chitinase 3-like 1 Quantikine ELISA Kit and Human Chitinase 3-like 1 Quantikine ELISA Kits according to the manufacturer’s instruction (R&D Systems, Minneapolis, MN). The human serum samples of normal objects and lung cancer patients were obtained from the Biosample Repository Core Facility (BRCF) of Fox Chase Cancer Center in Philadelphia (supported by NCI). The human serum samples were diluted to 1∶100 before assay.

### Western Blotting

For Stat3C-Flag fusion protein detection, CCSP-rtTA/(TetO)_7_-CMV-Stat3 bitransgenic mice were anesthetized and lungs were removed and homogenized in radioimmunoprecipitation assay (RIPA) buffer. Lung lysates (10 µg) were fractionated on a Novex® 4–20% Tris-Glycine Mini Gel (Invitrogen, Carlsbad, CA). After transferring to the polyvinylidene difluoride membrane (Bio-Rad, Hercules, CA), the membrane was blotted with 5% nonfat dry milk PBS and hybridized with rabbit anti-Flag antibody (Sigma, St Louis, MO). Following incubation with secondary antibody, the proteins were detected with chemiluminescent substrate (SuperSignal, Rockford, IL). For CHI3L1 protein detection, BALFs from doxycycline-treated or untreated CCSP-rtTA/(TetO)_7_-CMV-Stat3 bitransgenic mice were collected as described above and diluted with Laemmli sample buffer (Bio-Rad, Hercules, CA) at 1∶1, then heated in boiling water for 5 min. Gel electrophoresis and antibody hybridization were performed following the same procedure as above except that rat anti-mouse ChI3L1 primary antibody (1∶1,000, R&D, Minneapolis, MN) and goat anti rat IgG secondary antibody (1∶3000, Vector Laboratories, Burlingame, CA) were used. The protein bands were visualized with a Vectastain Elite ABC kit (Vector Laboratories, Burlingame, CA) following a procedure recommended by the manufacturer.

### Immunohistochemistry and Immunofluorescent Staining

For immunohistochemical staining, the lungs from doxycycline-treated or untreated CCSP-rtTA/(TetO)_7_-CMV-Stat3, CCSP-rtTA/(TetO)_7_-CMV-MMP12, CCSP-rtTA/(TetO)_7_-CMV-Api6, c-fms-rtTA/(TetO)_7_-CMV-Api6 and c-fms-rtTA/(TetO)_7_-CMV-MMP12 bitransgenic mice were inflated with a fixative solution (4% paraformaldehyde, 1 x PBS), dissected out, and stored in fixative at 4°C for 24 hours as previously described [8]. After fixation and embedding in paraffin, tissues were sectioned at 5-µm thick. Multiple sections from each lung were blocked with nonfat milk (4% in 1 x PBS) for 30 min., then incubated with rat anti-mouse CHI3L1 (1∶100; R&D Systems, Minneapolis, MN) at 4°C overnight. Tissue sections were washed and incubated with biotinylated secondary antibody goat anti-rat IgG. The signals were detected with a Vectastain Elite ABC kit (Vector Laboratories, Burlingame, CA) following a procedure recommended by the manufacturer.

For immunofluorescence staining, sections were hybridized with rat anti-mouse ChI3L1 antibody (R&D System) and rabbit anti-mouse F4/80 (abcam, Cambridge, MA) antibody as primary antibodies. A FITC-conjugated donkey anti-rat IgG (Santa Cruz, Dallas, Texas) and a Cy3-conjugated donkey anti-rabbit IgG (Jackson ImmunoResearch, West Grove, PA) at a dilution of 1/200 were used as the secondary antibodies. Sections were co-stained with DAPI. Slides were examined under a Nikon fluorescent microscope.

### Purification of Recombinant CHI3L1 Fusion Protein

The full-length murine *Chi3l1* coding region was amplified by PCR, and subcloned into the pEGX 4T-1 vector (GE life science, Pittsburgh, PA) at the NotI/BamHI restriction enzyme sites. CHI3L1 fusion protein was expressed in BL21 E. coli by 50 µM IPTG induction and purified using Glutathione Sepharose 4 Fast Flow kit (GE life science) according to the manufacture’s instruction.

### LLC Proliferation Assay and Annexin V assay

Lewis Lung Carcinoma (LLC) cells (5×10^3^/well) were seeded in 96-wells plate with culture medium (DMEM +10% FBS+1×PSA, Invitrogen). GST or GST CHI3L1 fusion protein (final concentration 100 ng/ml) was added to the culture medium. After 72 hours, cell numbers of proliferation were counted. For apoptotic assay, LLC cells (2x10^5^/well) were seeded in 6-wells plate with culture medium (DMEM +10% FBS+1×PSA, Invitrogen). GST or GST CHI3L1 fusion protein (final concentration 100 ng/ml) was added to the culture medium. After 72 hours, the apoptotic cell population was determined by fluorescein isothiocyanate-labeled-Annexin V (FITC-annexinV) (Apoptosis Detection Kit; BD PharMingen, Bedford, MA). LLC cells treated with GST or GST-CHI3L1 fusion protein were harvested and washed twice with PBS. After resuspension of the cells in Annexin V-binding buffer containing FITC-conjugated Annexin V (1∶20 dilution) and incubation at room temperature for 15 minutes, stained cells were analyzed by flow cytometry as soon as possible (within 1 hour).

### Statistical Analysis

The data were mean values of multiple independent experiments and expressed as the mean ± SD. ANOVA and Tukey’s method based on log-transformed concentration level were used to evaluate the significance of the differences. The log-transformation stabilized variance and made the statistical assumptions under ANOVA and Tukey’s method more plausible. ROC (Receiver Operating Characteristic) curve analysis was used to evaluate the diagnostic ability of CHI3L1, which is quantified by the area under the ROC curve (AUC). Analysis was performed using the pROC package in the R language [9]. Statistical significance level was set at p<0.05. Confidence Intervals (CIs) were also constructed for all estimates. For the ROC analysis when AUC is 1, the 95% CI has both lower and upper limits of 1, because AUC = 1 corresponds to total separation of two groups by CHI3L1 and the CI construction is based on the bootstrap procedure.

## Results

### Western Blot Analysis of CHI3L1 Expression in BALF of CCSP-rtTA/(TetO)_7_-CMV-Stat3C Mice

**Figure 1:**
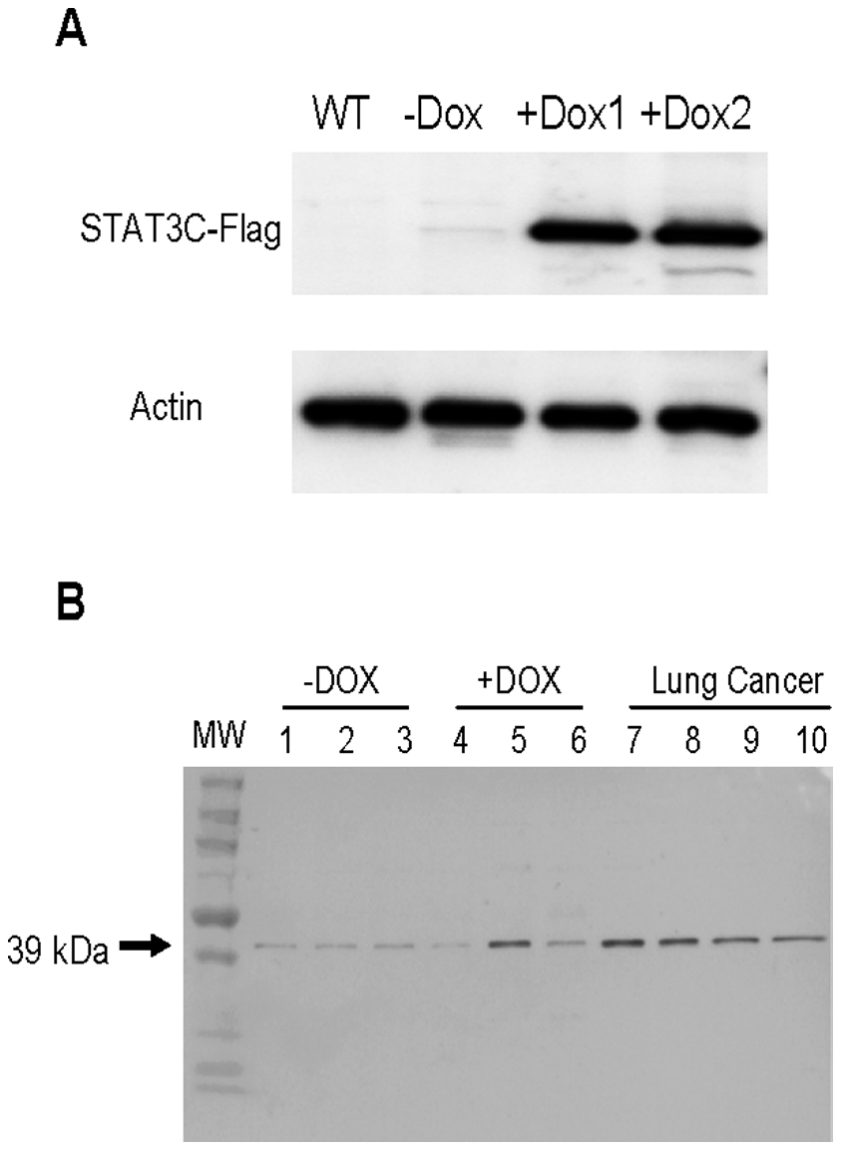
Western Blot analysis of Stat3C-Flag and CHI3L1 protein in CCSP-rtTA/(TetO)_7_-CMV-Stat3C mice. **A)** Expression of Stat3C-Flag in the whole lung of CCSP-rtTA/(tetO)7-CMV-Stat3C double-transgenic mice. Actin was used as control. WT: wild type mouse lungs; -Dox: doxycycline untreated bouble transgenic mouse lungs; +Dox1 & +Dox2: doxycycline treated mouse lungs. **B)** Expression of CHI3L1 protein in BALF of CCSP-rtTA/(TetO)_7_-CMV-Stat3C double-transgenic mice. Lane 1–3, doxycycline-untreated mice (Dox-); Lane 4–6, doxycycline-treated mice without showing lung tumor (Dox+); Lane 7–10, doxycycline-treated mice showing lung tumor (Lung Cancer).

As we reported previously, CCSP-rtTA/(TetO)_7_-CMV-Stat3C mice is a spontaneous lung tumor animal model. In this model, Stat3C-Flag fusion protein was highly inducible by doxycycline treatment (Figure 1A), which confirmed our previous observation by immunohistochemical staining in the lung [1]. Induction of Stat3C induced downstream genes (including CHI3L1), lung inflammation and adenocarcinoma [1], [6]. To test if CHI3L1 is a secretory protein in this mouse model, Western blot analysis was performed in BALF of CCSP-rtTA/(TetO)_7_-CMV-Stat3C mice. As shown in Figure 1, the CHI3L1 protein expression level was detectable in BALF of doxycycline-untreated mice (-DOX, Lanes 1, 2, and 3) as one band at 39 kDa molecular weight. In comparison, the CHI3L1 protein expression level was increased in one of three doxycycline-treated mice without tumor (+DOX, lane 5), indicating that CHI3L1 expression was elevated prior to tumor formation, and in all four doxycycline-treated CCSP-rtTA/(TetO)_7_-CMV-Stat3C mice with tumor (Lung cancer, lanes 7, 8, 9, and 10).

### CHI3L1 Protein Concentration in BALF and Serum from CCSP-rtTA/(TetO)_7_-CMV-Stat3C Mice

**Figure 2:**
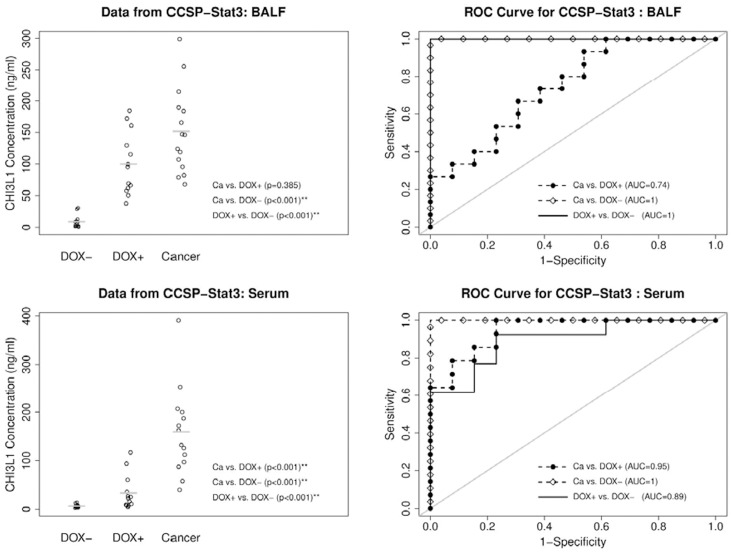
ELISA analyses of CHI3L1 protein in BALF and serum of CCSP-rtTA/(TetO)_7_-CMV-Stat3C bitransgenic mice. Left: CHI3L1 protein concentrations in BALF and serum of doxycycline-treated and untreated bitransgenic mice. Right: ROC (Receiver Operating Characteristic) curve analyses to determine the area under the curve (AUC). Dox-: doxycycline-untreated mice; Dox+: doxycycline-treated mice without showing lung tumor; Cancer, doxycycline-treated mice with lung tumor. Mean ± SD in BALF (n>13), DOX -: 8.8±10.0, DOX +: 100.0±49.0, Cancer: 151.8±67.3. Mean ± SD in serum (n>13), DOX -: 6.2±3.7, DOX +: 33.5±35.6, Cancer: 159.0±90.0. The gray lines represent the mean values.

For more quantitative analysis and accurate prediction, ELISA was used to determine the CHI3L1 concentration in BALF of wild type, doxycycline-untreated, treated without tumor, and treated with tumor CCSP-rtTA/(TetO)_7_-CMV-Stat3C mice. The average CHI3L1 concentration was 8.3±6.4 ng/ml in wild type mice, 8.8±10 ng/ml in doxycycline-untreated CCSP-rtTA/(TetO)_7_-CMV-Stat3C mice. In comparison, the average CHI3L1 concentration was increased more than 11-fold (100.0±49.0 ng/ml, p<0.001) in doxycycline-treated mice without tumor, further supporting that CHI3L1 expression was elevated prior to tumor formation, and was increased more than 17-fold (151.8±67.3 ng/ml, p<0.001) in doxycycline-treated mice with tumor (Figure 2, upper panel). The AUCs of the ROC curves are 1 (95% CI: 1–1, p<0.001) for discriminating doxycycline-treated mice with tumor vs. doxycycline-untreated mice, 1 (95% CI: 1–1, p<0.001) for doxycycline-treated mice without tumor vs. doxycycline-untreated mice, and 0.74 (95% CI: 0.56–0.93, p = 0.1) for doxycycline-treated mice with tumor vs. doxycycline-treated mice without tumor.

We further tested the CHI3L1 concentration in the blood. When compared with doxycycline-untreated mice (6.2±3.7 ng/ml), the average CHI3L1 concentration was increased more than 5-fold (33.5±35.6 ng/ml, p<0.001) in doxycycline-treated mice without tumor (prior tumor formation), and was increased more than 26-fold (159.0±90.0 ng/ml, p<0.001) in doxycycline-treated mice with tumor (Figure 2, lower panel). The AUCs of the ROC curves are 0.95 (95% CI: 0.87–1, p<0.001) for discriminating doxycycline-treated mice with tumor vs. doxycycline-treated without tumor mice, 1 (95% CI: 1–1, p<0.001) for doxycycline-treated mice with tumor vs. doxycycline-untreated mice, and 0.89 (95% CI: 0.77–1, p<0.001) for doxycycline-treated mice without tumor vs. doxycycline-untreated mice. This result clearly demonstrates that increase of the CHI3L1 protein concentration in BALF and blood is associated with Stat3C-induced inflammation and lung tumor. It seems that CHI3L1 protein increase occurred prior to spontaneous lung tumor formation in the CCSP-rtTA/(TetO)_7_-CMV-Stat3C mouse model, therefore potentially serves as biomarkers.

### CHI3L1 Protein Concentration in BALF and Serum of Regional Inflammation-induced Lung Tumor Animal Models

**Figure 3:**
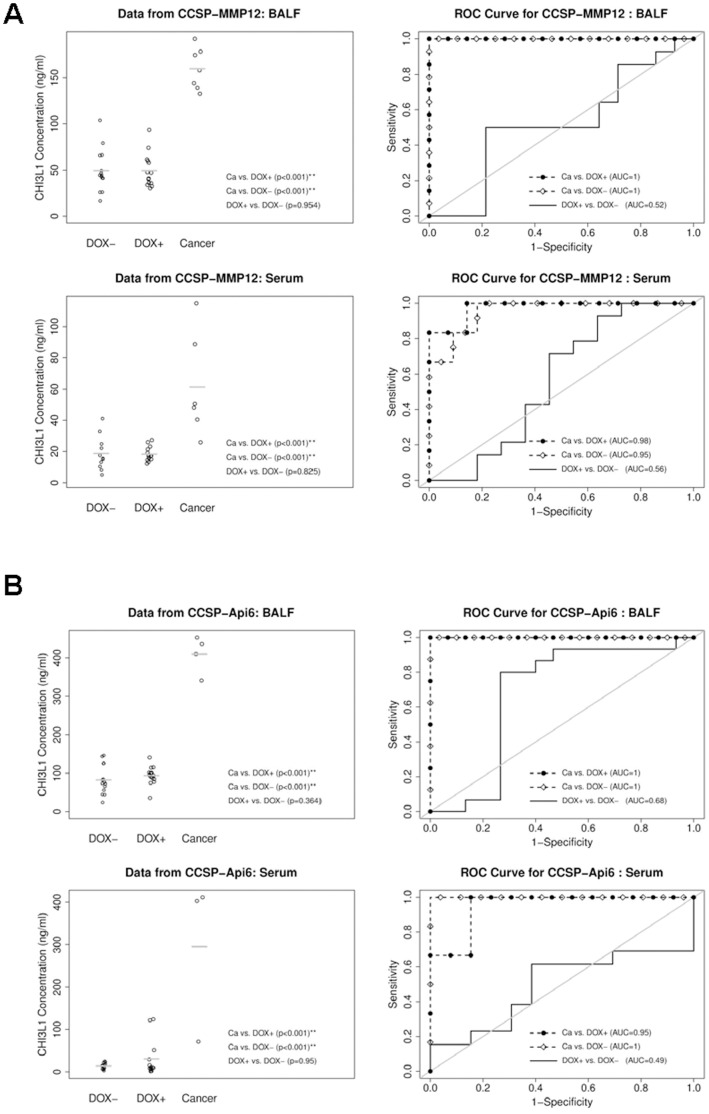
ELISA analyses of CHI3L1 protein in BALF and serum of regional inflammation-induced lung tumor mice. Left: CHI3L1 protein concentrations in BALF and serum of doxycycline-treated and untreated bitransgenic mice. Right: ROC (Receiver Operating Characteristic) curve analyses to determine the area under the curve (AUC). Dox-: doxycycline-untreated mice; Dox+: doxycycline-treated mice without showing lung tumor; Cancer, doxycycline-treated mice with lung tumor. **A)** CCSP-rtTA/(TetO)_7_-CMV-MMP12 mice. Mean ± SD in BALF (n>7), DOX -: 49.6±22.8, DOX +: 49.4±18.1, Cancer: 159.7±22.4. Mean ± SD in serum (n>7), DOX -: 18.6±10.9, DOX +: 18.2±4.6, Cancer: 61.4±33.5. The gray lines represent the mean values; **B)** CCSP-rtTA/(TetO)_7_-CMV-Api6 mice. Mean ± SD in BALF (n>3), DOX -: 82.4±37.1, DOX +: 93.8±23.0, Cancer: 409.8±49.2. Mean ± SD in serum (n>3), DOX -: 14.0±7.2, DOX +: 30.7±43.1, Cancer: 295.2±194.0. The gray lines represent the mean values.

To test if secretory CHI3L1 protein is up-regulated in a broader application, other inflammation-induced spontaneous lung tumor mouse models were tested. Matrix metalloproteinase 12 (MMP12) is a smoking-induced matrix metalloproteinase. Over-expression of MMP12 in lung alveolar type II epithelial cells induced local inflammation, emphysema and lung tumor [3]. In the CCSP-rtTA/(TetO)_7_-CMV-MMP12 spontaneous lung tumor mouse model, the average CHI3L1 concentration in BALF of doxycycline-treated mice with tumor was 3-fold higher (159.7±22.4 ng/ml) compared with that in BALF of doxycycline-untreated mice (49.6±22.8 ng/ml, p<0.001, Figure 3A, upper left panel). There was no significant difference between doxycycline-treated mice without tumor (49.4±18.1 ng/ml, p = 0.954) and doxycycline-untreated mice. A similar result was observed in the serum of MMP12-induced tumor mice (Figure 3A, lower left panel). ROC analysis indicated impressive capability of CHI3L1 concentration in discriminating the tumor group with the non-tumor group and the doxycycline-untreated group. The AUCs are 1 (95% CI: 1–1, p<0.001) in doxycycline-treated group with tumor vs. treated group without tumor and in doxycycline-treated group with tumor vs. doxycycline-untreated group, and 0.52 (95% CI: 0.29–0.75, p = 0.46) in doxycycline-treated group without tumor vs. dxycycline-untreated group (Figure 3A, upper right panel). A similar result was observed in the serum of CCSP-rtTA/(TetO)_7_-CMV-MMP12 spontaneous lung tumor mouse model (Figure 3A, lower right panel).

Apoptosis inhibitor 6 (Api6) is another pro-inflammatory molecule that induces inflammation and lung tumor when over-expressed in lung alveolar type II epithelial cells [5]. In the CCSP-rtTA/(TetO)_7_-CMV-Api6 spontaneous lung tumor mouse model, the average CHI3L1 concentration in BALF of doxycycline-treated mice with tumor was 409.8±49.2 ng/ml, compared with that in BALF of doxycycline-untreated mice (82.4±37.1 ng/ml, p<0.001, Figure 3B, upper left panel). Similarly, there was no significant difference between doxycycline-treated mice without tumor (93.8±23.9 ng/ml, p = 0.364) and doxycycline-untreated mice. A similar result was also observed in the serum of Api6-induced tumor mice (Figure 3B, lower left panel). The AUCs from the ROC analysis are 1 (95% CI: 1–1, p<0.001) in doxycycline-treated group with tumor vs. doxycycline-treated group without tumor and in doxycycline-treated group with tumor vs. doxycycline-untreated group, and 0.68 (95% CI: 0.45–0.90, p = 0.22) in doxycycline-treated group without tumor vs. doxycycline-untreated group (Figure 3B, upper right panel). A similar result was also observed in the serum of CCSP-rtTA/(TetO)_7_-CMV-Api6 spontaneous lung tumor mouse model, Figure 3B, lower right panel).

### CHI3L1 Protein Concentration in BALF and Serum of Systemic Inflammation-induced Lung Tumor Animal Models

**Figure 4:**
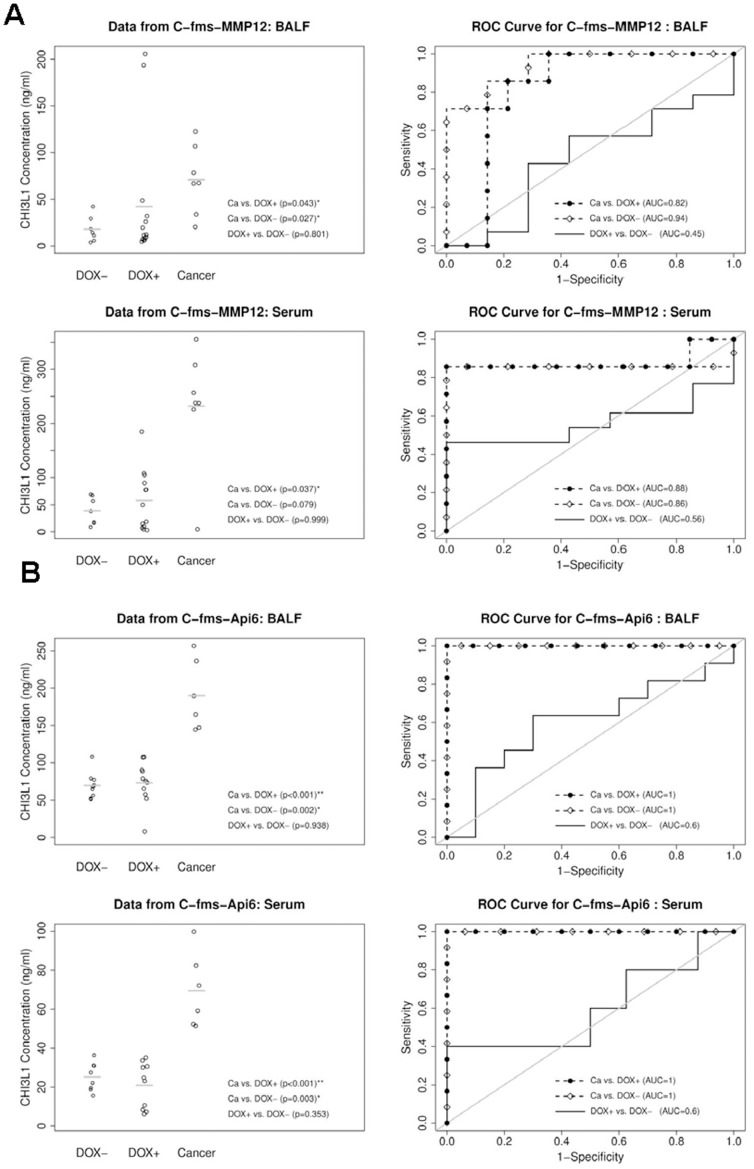
ELISA analyses of CHI3L1 protein in BALF and serum of systemic inflammation-induced lung tumor mice. Left: CHI3L1 protein concentrations in BALF and serum of doxycycline-treated and untreated bitransgenic mice. Right: ROC (Receiver Operating Characteristic) curve analyses to determine the area under the curve (AUC). Dox-: doxycycline-untreated mice; Dox+: doxycycline-treated mice without showing lung tumor; Cancer, doxycycline-treated mice with lung tumor. **A)** c-fms-rtTA/(TetO)_7_-CMV-MMP12 mice. Mean ± SD in BALF (n>7), DOX -: 17.7±13.8, DOX +: 42.1±67.9, Cancer: 70.9±36.5. Mean ± SD in serum (n>7), DOX -: 39.1±25.6, DOX +: 57.7±55.5, Cancer: 232.3±110.6. The gray lines represent the mean values; **B)** c-fms-rtTA/(TetO)_7_-CMV-Api6 mice. Mean ± SD in BALF (n>6), DOX -: 69.6±16.6, DOX +: 73.2±28.1, Cancer: 189.9±47.2. Mean ± SD in serum (n>6), DOX -: 25.2±7.3, DOX +: 21.0±11.7, Cancer: 69.5±19.1. The gray lines represent the mean values.

In contrast to lung regional inflammatory animal models, when MMP12 or Api6 was over-expressed in myeloid cells, systemic inflammation is initiated from malformation and malfunction of myelopoiesis from the bone marrow. Lung tumor formation was observed in both MMP12 and Api6 bitransgenic mouse models [2], [4]. This represents a different cellular mechanism for lung cancer formation. In the c-fms-rtTA/(TetO)_7_-CMV-MMP12 spontaneous lung tumor mouse model, the average CHI3L1 concentration in BALF of doxycycline-treated mice with tumor (70.9±36.5 ng/ml) was 4-fold increase in comparison with that in BALF of doxycycline-untreated mice (17.7±13.8 ng/ml, p = 0.027, Figure 4A, upper left panel). Doxycycline-treated mice without tumor (42.1±67.9 ng/ml, p = 0.043, Figure 4 A) also showed the increased CHI3L1 concentration compared with doxycycline-untreated mice. A similar result was observed in the serum of c-fms-rtTA/(tetO)_7_-CMV-MMP12 spontaneous lung tumor mouse model with tumor (Figure 4, lower left panel), in which the average CHI3L1 concentration was more than 6 times higher in tumor mice than that of doxycycline-untreated control mice. The AUCs from the ROC analysis were 0.82 (95% CI, 0.62–1, p = 0.05) in doxycycline-treated group with tumor vs. doxycycline-treated group without tumor, 0.94 (95% CI, 0.82–1, p<0.001) in doxycycline-treated group with tumor vs. doxycycline-untreated group, and 0.45 (95% CI, 0.17–0.73, p = 0.57) in doxycycline-treated group without tumor vs. doxycycline-untreated group (Figure 4A, upper right panel). Similar results were observed for serum samples of c-fms-rtTA/(tetO)_7_-CMV-MMP12 spontaneous lung tumor mouse model (Figure 4A, lower right panel).

In the c-fms-rtTA/(TetO)_7_-CMV-Api6 spontaneous lung tumor mouse model, the average CHI3L1 concentration in BALF of doxycycline-treated mice with tumor (189.6±47.2 ng/ml) was ∼3-fold higher than that in BALF of doxycycline-untreated mice (69.6±16.6 pg/ml, p = 0.002, Figure 4B, upper left panel). Similarly, there was no significant difference between doxycycline-treated mice without tumor (73.2±28.1 ng/ml, p<0.001) and doxycycline-untreated mice. A similar result was also observed in the serum of Api6-induced tumor mice (Figure 4B, lower left panel), in which the average CHI3L1 concentration was 3 times higher in tumor mice than that of doxycycline-untreated control mice. The AUCs from the ROC analysis are 1 in doxycycline-treated group with tumor vs. doxycycline-treated group without tumor, 1 in doxycycline-treated group with tumor vs. doxycycline-untreated group, and 0.6 (95% CI, 0.34–0.86, p = 0.35) in doxycycline-treated group without tumor vs. untreated group (Figure 4B, upper right panel). Similar results were observed for serum samples of c-fms-rtTA/(TetO)_7_-CMV-Api6 spontaneous lung tumor mouse model (Figure 4B, lower right panel).

### Lung Immunohistology

**Figure 5:**
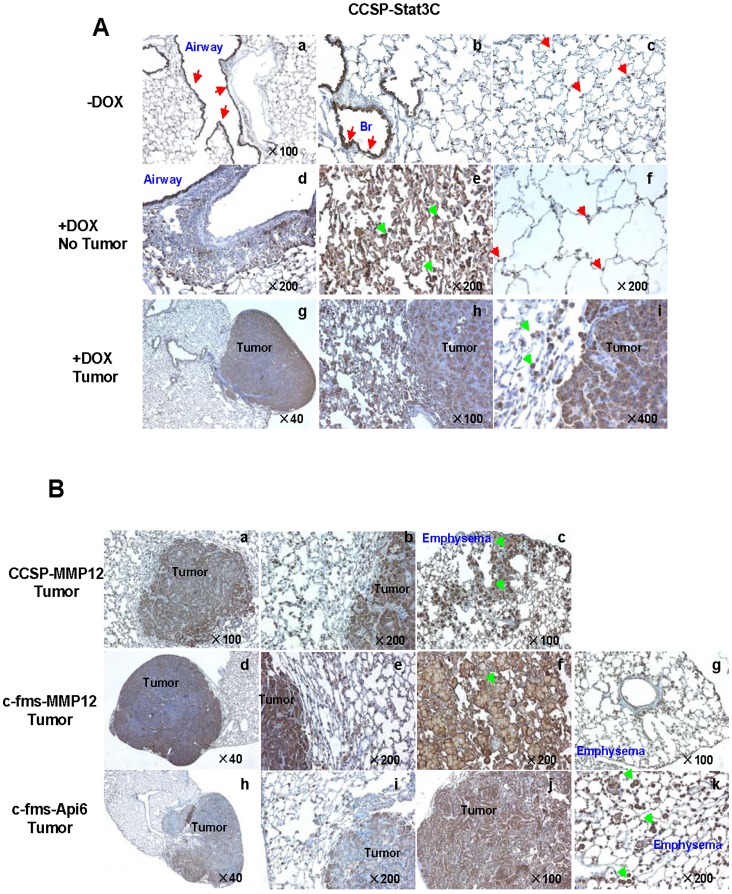
Immunohistochemical staining of CHI3L1 protein in the lung of inflammation-induced tumor mice. **A)** Immunohistochemical staining in the lungs of doxycycline-treated or untreated CCSP-rtTA/(TetO)_7_-CMV-Stat3C mice; **B)** Immunohistochemical staining of tumor regions in the lungs of doxycycline-treated CCSP-rtTA/(TetO)_7_-CMV-MMP12 mice, c-fms-rtTA/(TetO)_7_-CMV-MMP12 mice, and c-fms-rtTA/(TetO)_7_-CMV-Api6 mice. -Dox: doxycycline-untreated mice; +Dox: doxycycline-treated mice.

**Figure 6:**
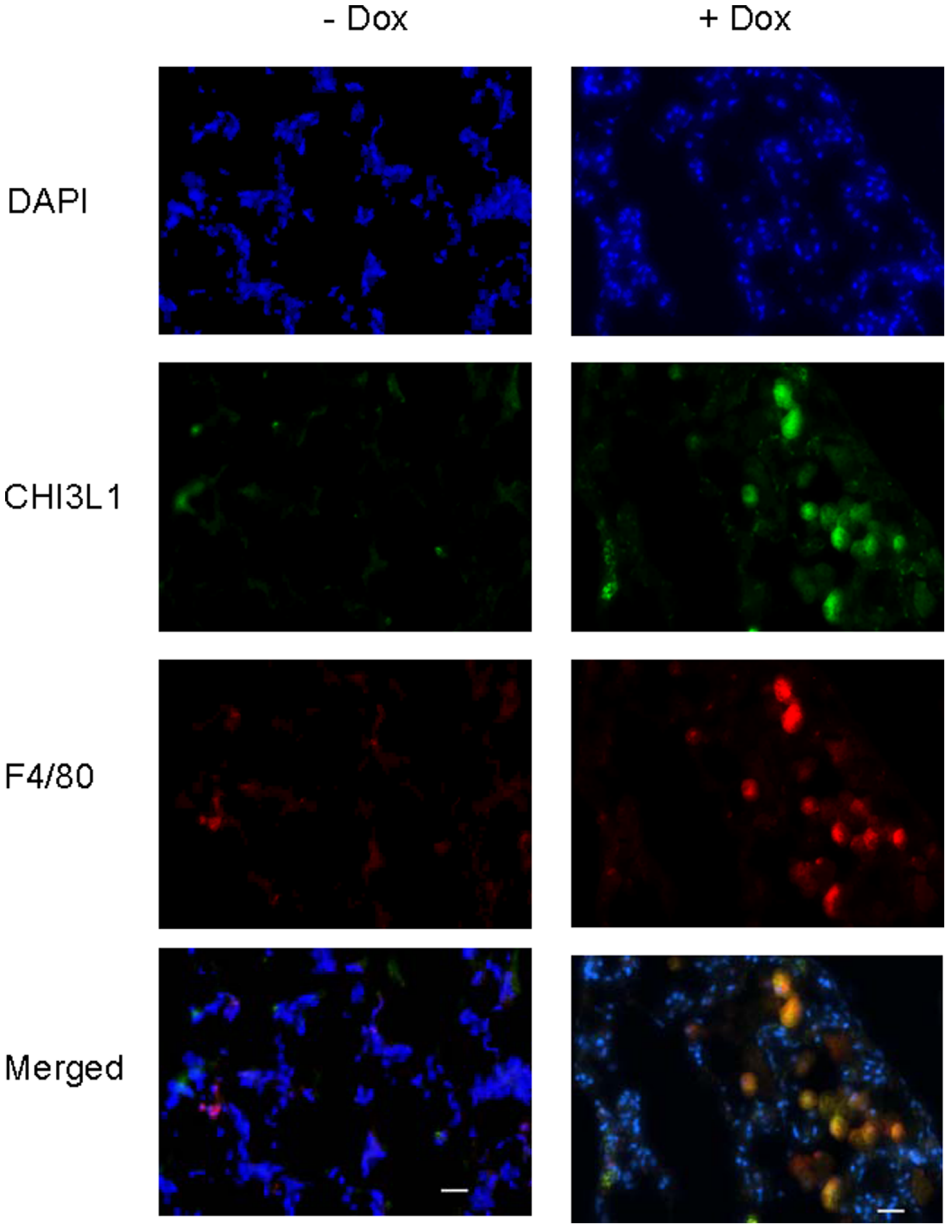
Immunofluorescent co-staining of CHI3L1 and F4/80 in the lung of CCSP-rtTA/(TetO)_7_-CMV-Stat3C mice. CCSP-rtTA/(TetO)_7_-CMV-Stat3C bitransgenic mice were treated (+DOX) or untreated (-DOX) with doxycycline. Immunofluorescent staining of lung sections was performed using anti-CHI3L1 and F4/80 antibodies. Co-localization of both staining was observed in the merged picture. Bar represents 20 µm.

It is important to locate where CHI3L1 is expressed in the lung of above spontaneous lung tumor mouse models. Lung tissue sections from doxycycline-untreated mice, -treated without tumor, and -treated with tumor mice were immunohistochemically stained with anti-CHI3L1 antibody. In doxycycline-untreated CCSP-rtTA/(TetO)_7_-CMV-Stat3C mice, CHI3L1 was mainly expressed in epithelial cells along the conducting airways (Figure 5 A a), distal bronchiolar epithelial cells (Figure 5A b), and alveolar type II epithelial cells (Figure 5 A c) (red arrows). CHI3L1 was also expressed in alveolar macrophages. In doxycycline-treated CCSP-rtTA/(TetO)_7_-CMV-Stat3C mice when inflammation was induced but with no tumor appearance, some areas showed tissue remodeling (Figure 5A e) and emphysema (Figure 5A f). Almost all infiltrated macrophages were stained positively with CHI3L1 (Figure 5A e, green arrow). In doxycycline-treated CCSP-rtTA/(TetO)_7_-CMV-Stat3C mice with tumor appearance, CHI3L1 expression was detected in tumor cells and surrounding macrophages (Figure 5A g, h, i). Co-localization of CHI3L1 in alveolar macrophages was further confirmed in the lung of doxycycline-treated bitransgenic mice with anti-CHI3L1 and anti-F4/80 antibodies in immunofluorescent staining. A representative result from CCSP-rtTA/(TetO)_7_-CMV-Stat3C mice was presented in Figure 6.

These observations were largely repeatable in other MMP12 and Api6-induced spontaneous lung tumor mouse models. Regardless of lung tumor initiated from alveolar type II cells or myeloid cells, CHI3L1 was over-expressed in tumor cells of these MMP12 and Api6-induced spontaneous lung tumor mouse models. Macrophages surrounding emphysema (Figure 5B c, g, k) and tumor cells (Figure 5B b, e, f, i) were also intensely stained by anti-CHI3L1 antibody (green arrow). This may explain why the CHI3L1 concentration was higher in both BALF and blood of tumor mice.

### CHI3L1 Stimulation on Cancer Cells

**Figure 7:**
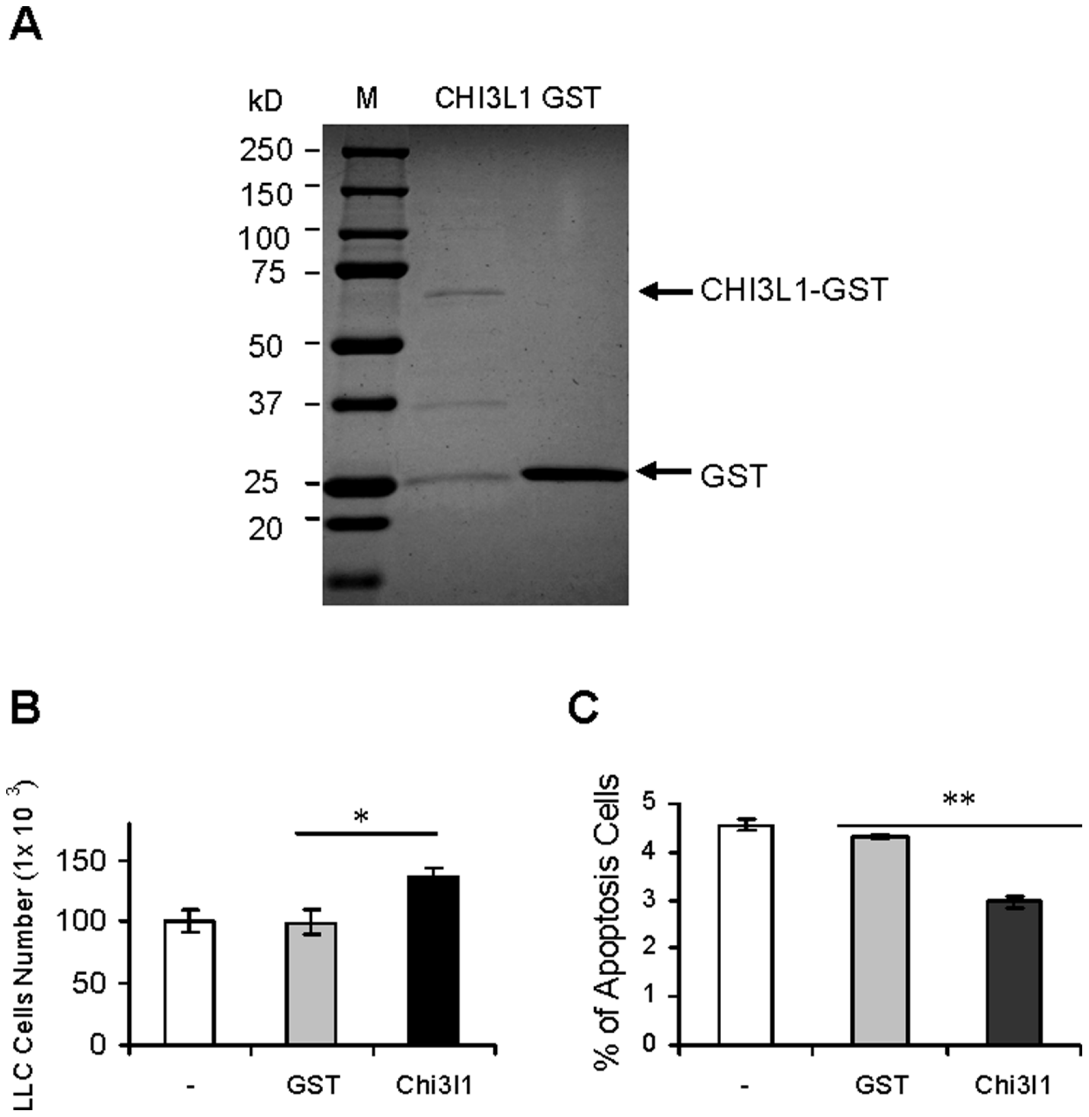
Stimulatory effect of CHI3L1 on tumor cells *in vitro*. **A)** Purity of recombinant CHI3L1-Flag fusion protein (upper arrow); **B)** LLC cell proliferation treated with GST or CHI3L1-GST fusion protein; **C)** The apoptotic activity of LLC cells treated with GST or CHI3L1-GST fusion protein by Annexin V labeling assay. *, p<0.05, **, P<0.01.

To test if CHI3L1 possesses the stimulatory effect on lung cancer cells, recombinant CHI3L1-GST fusion protein and GST protein were prepared and purified from bacteria (Figure 7A). Recombinant CHI3L1-GST fusion protein or control GST protein was incubated with LLC cells. CHI3L1-GST fusion protein significantly stimulated cancer cell proliferation compared with that of GST control protein (Figure 7B). The apoptotic activity of CHI3L1-GST fusion protein treated LLC cells was reduced compared with that of GST control protein (Figure 7C). This supports a concept that CHI3L1 stimulates lung cancer proliferation and growth.

### CHI3L1 Protein Concentration in the Serum from Human Lung Cancer

**Figure 8:**
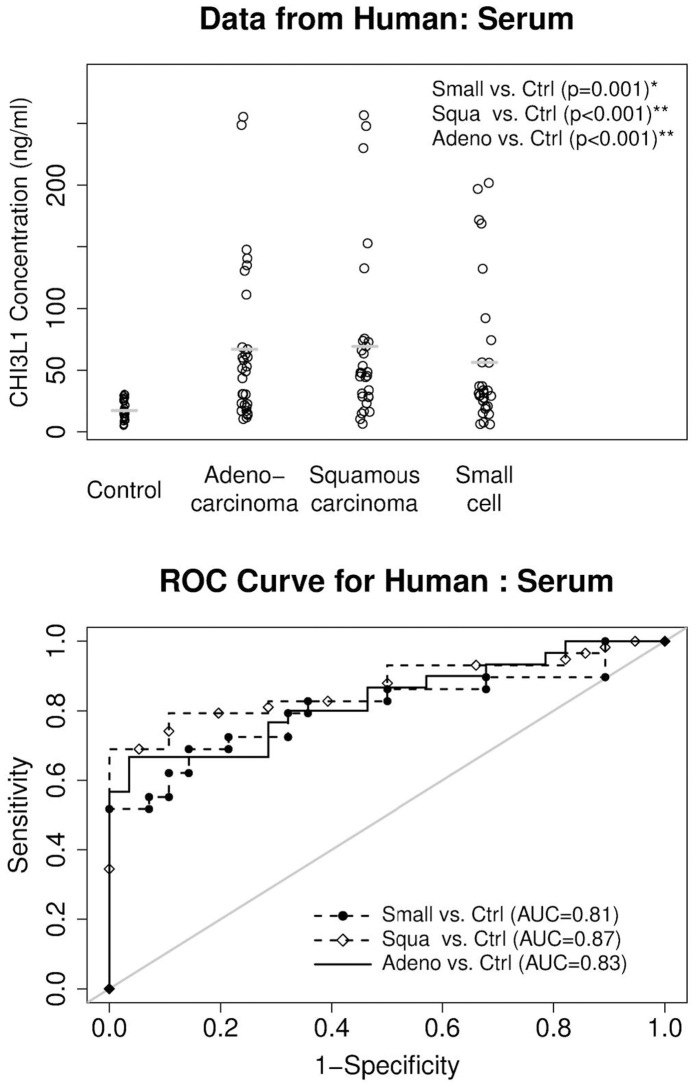
ELISA analysis of CHI3L1 protein in serum of human lung cancer patients. Top: CHI3L1 protein concentrations in human serum. Bottom: ROC (Receiver Operating Characteristic) curve analysis to determine the area under the curve (AUC). Mean ± SD in control (n>30): 17.3±7.8. Mean ± SD in adenocarcinoma (n>30): 66.9±64.8. Mean ± SD in squamous carcinoma (n>30): 69.3±69.0. Mean ± SD in small cell cancer (n>30): 56.3±58.8. The gray lines represent the mean values. Control: Normal human without cancer.

Since CHI3L1 protein concentration correlates very well with tumor occurrence in both regional inflammation and systemic inflammation-initiated spontaneous lung tumor mouse models, it is intriguing to determine if this observation can be repeated in humans. Approximately 30 serum samples from human patients of lung adenocarcinoma, lung squamous carcinoma and lung small cell cancer were tested by ELISA. Compared with human serum samples without any cancer (normal), the CHI3L1 protein concentration was significantly elevated in human serum samples from all categories of lung cancer patients (Figure 8, upper panel). The AUCs are 0.83 (95% confidence interval, 0.69–0.92, P = 0.005) for adenocarcinoma vs. Control, 0.87 (95% confidence interval, 0.77–0.97, P<0.001) for squamous carcinoma vs. Control, and 0.81 (95% confidence interval, 0.72–0.94, P = 0.001) for small cell carcinoma vs control (Figure 8, lower panel).

## Discussion

CHI3L1 was first identified as a downstream gene of Stat3 in the CCSP-rtTA/(tetO)_7_Stat3C spontaneous lung tumor mouse model by Affymetrix GeneChip microarray [1]. Its secretion was detectable in BALF of doxycycline-untreated mice (normal control) (Figure 1 B), implicating a requirement of this protein under the normal physiological condition. Its spatial expression was restrictive to alveolar type II epithelial cells and conducting airway epithelial cells, as well as alveolar macrophages in the normal mouse lung (Figure 5A a-c, Figure 6). During inflammatory status, after Stat3C overexpression by doxycycline induction, the concentration of CHI3L1 was elevated in BALF of tumor-forming CCSP-rtTA/(tetO)_7_Stat3C bitransgenic mice (Figure 1B, lane 5, Figure 2). This is tightly associated with strong tumor cell expression of CHI3L1 in the lung (Figure 5A g-i). Even in the non-tumor-forming mice, CHI3L1 expression was highly induced in inflammatory cells of the lung (Figure 5A d-e, Figure 6). This often accompanied with emphysema (Figure 5A f). Both tumor cells and inflammatory cells contribute to CHI3L1 protein elevation in BALF of doxycycline-treated CCSP-rtTA/(tetO)_7_Stat3C bitransgenic mice. Interestingly, the CHI3L1 protein concentration was also elevated in the blood serum of doxycycline-treated CCSP-rtTA/(tetO)_7_Stat3C bitransgenic mice (Figure 2, lower panel). At least the myeloid population contributes to the increase of the CHI3L1 protein concentration in the blood of doxycycline-treated CCSP-rtTA/(tetO)_7_Stat3C bitransgenic mice. As we reported previously, MDSCs play an important role to lung cancer formation in the CCSP-rtTA/(tetO)_7_Stat3C bitransgenic mouse model [6].

In other regional inflammation-induced lung spontaneous tumor mouse models, overexpression of MMP12 and Api6 in alveolar type II epithelial cells results in Stat3 activation (increased phosphorylation at Y705) in lung epithelial cells [3] [5]. Similar to that observed in CCSP-rtTA/(TetO)_7_-CMV-Stat3C bitransgenic mice, the increased CHI3L1 protein concentration was observed in BALF and serum of doxycycline-treated CCSP-rtTA/(TetO)_7_-CMV-MMP12 and CCSP-rtTA/(TetO)_7_-CMV-Api6 bitransgenic mice with tumor. Unlike that observed in CCSP-rtTA/(TetO)_7_-CMV-Stat3C bitransgenic mice, no increase of the CHI3L1 protein concentration was observed in non-tumor mice even after doxycycline treatment (Figure 3). The study was also repeated in two systemic inflammation-induced tumor models (Figure 4), in which MMP12 and Api6 were overexpressed in myeloid cells of c-fms-rtTA/(TetO)_7_-CMV-MMP12 and c-fms-rtTA/(TetO)_7_-CMV-Api6 bitransgenic mice [2], [4]. Stat3 was activated in lung epithelial cells of both systemic inflammation-induced tumor models [2], [4]. Based on these extensive studies in multiple inflammation-induced lung tumor mouse models, it further proves that Stat3 and its downstream genes are involved in lung tumorigenesis. The products of these genes can serve as potential biomarkers for lung cancer prediction and verification.

Indeed, the CHI3L1 protein concentration in the serum of human patients with several types of lung cancer was significantly elevated (Figure 8). A report has shown that elevated pretreatment serum concentration of CHI3L1 serves as an independent prognostic biomarker for poor survival in patients with metastatic non-small cell lung cancer [10]. The elevated level of CHI3L1 is also observed in chronic obstructive pulmonary disease (COPD) [11], and plays a role in the pathogenesis of cigarette smoke-induced inflammation and emphysema [12]. This is similar to what we observed in CCSP-rtTA/(tetO)_7_Stat3C bitransgenic mice (Figure 5f). COPD is a disease highly associated with smoking. The human COPD population is a high risk population to develop lung cancer. It is important to keep in mind that the CHI3L1 upstream Stat3 oncogene is up-regulated in both human lung cancer and COPD [7]. Additionally, higher expression levels of MMP12 can be induced by chronic smoking [12], [13]. Since MMP12 plays a critical role in emphysema to lung cancer transition [3], [4], increased CHI3L1 expression may play an important role to facilitate this pathogenic process by promoting inflammation as demonstrated in Figure 3A and 4A. CHI3L1 may serve as a new target for treatment of COPD/emphysema and lung cancer. As a matter of fact, recombinant CHI3L1 fusion protein directly stimulated LLC proliferation and growth (Figure 7).

In summary, this report shows that CHI3L1 is a potential biomarker for inflammation-induced lung cancer prediction in animal models, especially in Stat3-induced cancer. Although CHI3L1 is a useful biomarker, it is necessary to use multiple biomarkers as a panel for more accurate prediction and verification of lung cancer, as increase of CHI3L1 expression has been shown in other inflammation-related mouse models [14] [15], [16], [17], [18]. We have recently identified additional secretory proteins that are products of Stat3 downstream genes as biomarkers for lung cancer for this purpose. After thoroughly characterizing each one of these proteins in our lung cancer animal models and human tissues, we will combine them as a panel to discriminate cancer-related inflammation from non-cancerous inflammation, as well as lung cancer from other types of cancers. The novelty of this work is three folds: 1) Although it has been demonstrated that CHI3L1 is associated with human lung cancer, no study has been done in lung cancer animal models. We are the first group to study CHI3L1 using multiple spontaneous lung cancer animal models. 2) Our work for the first time revealed a relationship between CHI3L1 with Stat3, MMP-12, and Api6-induced spontaneous lung tumor formation. This provides a significant mechanistic insight into the inflammation-induced lung cancer formation; 3) The animal models used in our work are unique, which give us a unique opportunity to study the basic and clinical directions of CHI3L1. These animal models will facilitate identification of additional biomarkers to predict and verify lung cancer under various pathogenic conditions, which normally cannot be done in humans.
